# Alleviating cross-cultural challenges of Indian subcontinent students: University staff perspectives

**DOI:** 10.1007/s11233-022-09094-8

**Published:** 2022-07-12

**Authors:** Monika Kansal, Ritesh Chugh, Anthony Weber, Stephanie Macht, Robert Grose, Mahsood Shah

**Affiliations:** 1 School of Business and Law, Central Queensland University, Queensland, Australia; 2 School of Engineering and Technology, Central Queensland University, Queensland, Australia; 3 Swinburne University of Technology, Sydney, Australia

**Keywords:** Cross-cultural, Subcontinent, International students, Teaching, Support

## Abstract

The growth in student numbers from the Indian subcontinent countries has increased exponentially in the Australian higher education system over the past decade. Unfortunately, this growth has not been accompanied by initiatives to address the distinctive cross-cultural challenges faced by this cohort. This paper seeks to identify academic and professional staff perceptions of how they can help alleviate the social and academic challenges faced by subcontinent students. Thematic analysis of multiple focus group interviews established a range of simple initiatives that university staff and decision-makers could introduce to alleviate these challenges. Academic and professional staff should clearly and repeatedly articulate course, assessment and integrity expectations and make use of pre-arrival and orientation interactions. The pedagogic environment should be customised to subcontinent students in the classroom, and written teaching materials should be supplemented with engaging videos, ideally subtitled. The introduction of cross-cultural awareness training for academic and professional staff would improve the learning experience of subcontinent students. Adequate resourcing of academic skills and English language support and mental health support services also emerged as valuable initiatives. The cross-cultural awareness training for academic and professional staff should also be customised to their specific roles.

## Introduction

International students represent a significant resource for Australia and Australian universities (Newton et al., 2021; Ross, 2020; Welch, 2020). In 2019, there were 442,219 international students enrolled in Australian higher education institutions – this number has been growing at an average rate of about 10% per annum over the last five years (Department of Education, Skills and Employment 2020a). Most international students in Australia study management, commerce, engineering, information technology or other technology-related subjects. Most international students hail from China and the Indian subcontinent (Department of Education, Skills and Employment 2020b). COVID-19 has worked as a circuit breaker in the business model of Australian universities as the Australian Government has restricted universities from physically accepting students from international markets such as China, Nepal, and India. These restrictions have severely impacted their revenue streams (Ross, 2020). Although this study was completed in pre-COVID-19 times, the findings are still relevant as, from 15 December 2021, fully vaccinated international students are able to travel to Australia to study without needing to apply for a travel exemption (Department of Home Affairs, 2021).

Given the significant growth in international student numbers in the pre-COVID-19 era, academics and professional staff in higher education institutions must be capable of addressing the distinctive needs and cross-cultural challenges faced by international students. The literature highlights a range of challenges faced by international students which can be broadly grouped into academic challenges (including meeting visa requirements, language barriers and different educational requirements, teaching technologies, learning styles and learning practices) and non-academic/social challenges (including culture shock, economic barriers, lack of family support and different cultural values) (e.g., Cetinkaya-Yildiz et al., 2011; Newton et al., 2021).

Although these challenges are well documented in the literature, the relevant body of knowledge displays significant weaknesses, summarised as two overlapping gaps in the international student education literature. The first gap derives from the under-representation of studies focusing on students from the Indian subcontinent, while the second gap lies in the lack of research from a university staff perspective.

In terms of the first identified gap, extant literature tends to focus on ‘international students’, as one group (e.g., Trice 2003; Gunawardena & Wilson, 2012), and much of this literature concentrates on Chinese students (e.g., Jin and Cortazzi 2001; Teo & Arkoudis 2019). Students from the Indian subcontinent, a reasonably heterogeneous subgroup of international students (Atri & Sharma, 2006), are ignored in the literature. Ignoring this subgroup is a major omission as a substantial proportion of international students, especially in countries like Australia, hail from the Indian subcontinent. In 2019, 20.5% of international higher education students in Australia came from India (90,763 students), and a further 7.8% came from Nepal (34,632 students) (Department of Education, Skills and Employment 2020b).

The second identified gap refers to the literature’s over-reliance on the student perspective. While it is naturally essential to explore the perspective of those who are facing the challenges, it is equally important to acknowledge that other individuals may have in-depth insights into students’ experiences and challenges and can provide useful additional viewpoints to better understand the challenges and how to support students in overcoming these challenges. Given that academics and professional staff work closely with international students, they are ideally placed to understand many of the challenges faced by this cohort of students, and they also have experience supporting students through their struggles (Newton et al., 2021; O’Reilly et al., 2013). Newton et al., (2021) explored the challenges and opportunities for improving the health and well-being of international students and reported the perceptions of a small sample of professional staff in an Australian university. However, the study was limited to the perceptions of professional staff and did not use a representative sample covering all roles supporting the well-being of international students within the sample university from a cultural perspective. This study fills the gap by taking a more extensive and comprehensive sample of both professional and academic staff. Unlike previous literature exploring international students’ wider health and well-being issues as one cohort (Newton et al., 2021; O’Reilly et al. 2013), this study presents an exclusive focus on students from the Indian subcontinent and answers specific questions relating to alleviating their cross-cultural challenges. There is a dire need and marked paucity of research exploring the perceptions and experiences of university staff in their endeavours to support international students and alleviate (some of) the challenges identified above (Bird, 2017; Newton et al., 2021; O’Reilly et al. 2013). This study is timely and relevant in the present context where the Australian higher education sector reflects higher levels of engagement with students from Asia (Welch, 2020).

This paper is exploratory in nature, as it aims to add knowledge to address the two identified gaps in the extant literature. Specifically, this paper explores the viewpoints and experiences of university staff in the context of supporting international students from the Indian subcontinent. To achieve its purpose, the paper aims to address the following research question:

*Based on the experience of staff working with subcontinent students, what initiatives can be implemented in universities to help alleviate the cross-cultural challenges faced by Indian subcontinent students?*

The remainder of this paper provides an overview of the scarce literature on the cross-cultural challenges faced by students from the Indian subcontinent and outlines the role university staff can play to alleviate these challenges. The paper then discusses the data collection and analysis approaches before detailing the thematic findings from the rich, qualitative data. Finally, the paper concludes with some recommendations for higher education institutions and further research.

## Cross-cultural challenges for subcontinent students

Students from the Indian subcontinent face a range of cross-cultural challenges. One of the key challenges the literature discusses in terms of international students is the language barrier faced by most international students’ (Jenkins & Galloway, 2009; Newton et al., 2021). In fact, the literature suggests that students themselves consider language barriers to be their most significant challenge, which, in turn, can lead to issues such as plagiarism, a lack of understanding of the learning and teaching methods and poor interpretation of material (Bird, 2017; Vinther and Slethau 2015). Even if language was not an issue, international students often struggle with the educators’ use of local examples and case studies in teaching, particularly if taught alongside domestic students.

A significant cross-cultural challenge for students studying in other countries resides in the differences in teaching and learning styles between their home and host countries (Sakurai et al., 2016), and they are not well prepared to face the ‘gap’ between their expectations and reality (Kingston & Forland, 2008). Australian higher education mainly relies on a student-centred and deep learning approach, which values critical thinking, independent study, and discussions with teachers as facilitators (Bird, 2017). This is in stark contrast to the educational traditions in Indian subcontinent countries, which rely on teacher-focused and surface approaches to learning, and where the teacher is considered a ‘guru’ who provides the student with knowledge and should not be questioned. Students coming from this type of educational environment rely on memorising and regurgitating the information provided by the teacher and often prefer to take a passive role in the classroom (Manikutty et al., 2007; Marambe et al., 2012). Adding to this passive learning style is a common approach observed in collectivist cultures like India, where students often prefer not to debate with or contradict fellow students to avoid loss of face (Manikutty et al., 2007).

A further impact of collectivistic approaches is that international students have strong desires to build interpersonal relationships, which they use to solve personal and academic problems (Shuter et al., 2018). The latter can be problematic as it may lead to collusion in assessments. Students from collectivist countries often have not encountered this form of academic misconduct in their home country as they were previously allowed, if not encouraged, to collaborate (Song-Turner, 2008).

A similar issue arises with other forms of academic dishonesty. For example, students from the Indian subcontinent have grown up in an educational culture where it is considered respectful to the author to repeat published work verbatim or construct arguments based on existing sources without citing these sources (Handa & Power, 2005; Song-Turner, 2008). Although students are generally informed of the importance of academic integrity, it is not uncommon for international students to intuitively use the learning and integrity approaches from their native culture (Duff & McKinstry, 2007). This approach is particularly evident during stressful times, such as those encountered when international students attempt to adapt to the new culture and learning environment, potentially alongside other factors, such as financial pressures, homesickness, and religious challenges (Atri & Sharma, 2006; Chirkov et al., 2008; O’Reilly et al. 2013), or challenges resulting from the students’ specific motivation to seek education in the first instance. For example, Birrell’s (2019) study of international students suggested their primary purpose for studying in Australia was to seek other visas, and their university study was considered a ‘necessary evil’, rather than the main focus.

## The role of university staff in supporting subcontinent students

The Higher Education Standards Framework requires higher education institutions to ensure that the educational needs of student cohorts are met (Tertiary Education Quality and Standards Agency [TEQSA] 2015). Nevertheless, the scant literature in this field suggests that many university staff do not fully understand students’ needs (Roberts & Dunworth, 2012). They also believe that international students should fully adapt to the host culture, a perspective referred to as the “colonial hangover” model of higher education (Wisker, 2000). On the other hand, there are staff members who acknowledge that working with international students requires *cultural synergy*, which requires the “*mutual effort from all participants to learn about, understand and appreciate others’ cultures and their interpretations of learning and reciprocally to learn with and from others*” (Jin and Cortazzi 2001).

University staff play an integral role in the education and support of all international students, including those from the Indian subcontinent. Some researchers propose that staff need to recognise international students’ characteristics and values and actively support them in their adjustment to the host country’s educational environment (Bird, 2017; Vinther and Slethau 2015). This requires educators, in particular, to understand how approaches to teaching and learning differ across cultures (Manikutty et al., 2007) and to acknowledge that they have an active role to play in supporting students’ transition to a new learning environment (Baeten et al., 2010).

Staff development is crucial for university employees to effectively manage a diverse student body, and better understand student needs, and subsequently reap benefits for international students, the institution, and themselves. Domestic students will also benefit from cross-cultural exposure (Vinther & Slethaug, 2015; Zhang & Dinh, 2017). Professional development is particularly pertinent for professional staff, such as advisors, who can have a significant impact on the student experience (Briggs & Pritchett, 2010), but who, according to the literature, appear to receive little training to support international students (Jacob et al., 2015; Zhang & Dinh, 2017).

## Methods

This paper focuses on staff experiences in alleviating the cross-cultural challenges faced by subcontinent students. The study collected qualitative data through semi-structured focus group interviews. Qualitative methods are best suited to projects that aim to better understand complex and under-researched phenomena by making sense of participants’ meanings and lived experiences (Powell & Single, 1996). Focus groups generate rich data by exploring respondents’ perceptions, experiences and divergent views through guided discussions with the researcher and other participants (Powell & Single, 1996). Aligned with the qualitative approach, this inductive study provides novel insights to conceptualise new issues in higher education research (Dicker et al., 2019; Kitzinger, 1994).

After obtaining ethics approval for the project, participants were recruited in the following manner. All academic staff (including casual staff) teaching on two metropolitan campuses within the business and engineering schools (the schools with the largest numbers of subcontinent students) and all professional staff located on the same campuses were approached via email invitations. Twenty-nine academic staff members and 21 full-time professional staff from diverse university areas (student services, learning and teaching support, counselling and advocacy, and international student support) volunteered to participate. Overall, the research team interviewed 50 staff members (n = 50) via eight focus groups (four professional and four academic staff). This number is in line with the recommendations from the literature that three to four focus groups are required to help identify dominant themes (Guest et al., 2017; Richter et al., 1991).

After an extensive literature review, interview guides were developed, and pilot tested on university peers to detect duplications, redundancies and ambiguous questions. The interview guides included questions about staff experience of subcontinent students’ cross-cultural challenges, reflections on their attempts at alleviating such challenges, and suggestions based on their own experience. The role of the focus group facilitators was to ask the questions, keep the conversation flowing by encouraging all participants to express their views, and ensure conversations remained on topic. On average, the focus groups lasted 72 min, and all interviews were audiotaped and transcribed verbatim by a professional transcriber. Further details regarding focus groups are presented in Table 1.

**Table 1:** Details of the focus group interviews

Staff	Duration of focus groups	Number of participants
Professional Staff_Group 1	1 h and 3 min	5
Professional Staff_Group 2	1 h and 14 min	5
Professional Staff_Group 3	58 min	4
Professional Staff_Group 4	1 h 21 min	7
Total	**276 min**	**21**
Academic Staff_Group 1	1 h 51 min	9
Academic Staff_Group 2	1 h and 8 min	6
Academic Staff_Group 3	1 h and 22 min	8
Academic Staff_Group 4	37 min	6
Total	**298 min**	**29**

The response data were coded as FG1 (Focus Group1), AS (Academic Staff), PS (Professional Staff) or P1 (Participant 1) to de-identify the data. To maintain anonymity, the personal details of participants were not collected, and only aggregated results were presented. While analysing data, we considered individual focus groups as a ‘unit of analysis’ rather than as an individual participant. This approach ensured that we were not presenting the dominant voice of a few outspoken participants. The facilitation of each focus group was carried out by experienced moderators who encouraged all participants to express their views (Smithson, 2000).

To minimise inter-rater bias, the transcripts were independently coded by two researchers. Codes were iteratively compared to one another and across focus groups, allowing the researchers to group multiple codes into broader, mono-thematic categories and establish whether codes occurred as single instances (these were removed) or common themes (these were retained as common findings across the study sample). NVivo was used for the thematic analysis, whereby coding was refined, new themes were added, and earlier themes were revised as data analysis progressed. Emerging themes were again iteratively compared to one another and across the data set to ensure all potential themes had been identified. Analysis and interpretation were completed as per Gale et al.’s (2013) six-stage analysis framework – transcription; initial reviewing of the interview data using interview guides as a starting point; coding by comparing and contrasting viewpoints; developing a working analytical framework; applying the analytical framework; and final interpretation of the data.

## Results and discussion

Analysis of the focus group discussions suggested that the ‘colonial hangover’ model of higher education, whereby the onus on acculturation is solely on the student (Wisker, 2000) is unsuitable as all participating staff considered themselves to have a significant role to play in alleviating subcontinent students’ cross-cultural challenges. However, they also acknowledged that there is another university stakeholder that also needs to play a role in this endeavour: university decision-makers. Accordingly, the discussion that follows is separated into two distinct sections. First, we provide an overview of staff perspectives and experiences regarding strategies decision-makers could take to help reduce the challenges faced by subcontinent students. Secondly, we cover the staff perceptions regarding actions that university administrators should initiate to alleviate such challenges. In both sections, we present specific initiatives that the participating staff recommended and illustrate these with the help of quotes from the rich data. Since some of these quotes refer to ‘international students’, it is important to stress that whenever a participant used this terminology in their response the focus group facilitators confirmed the focus of this study is students from the Indian subcontinent. Therefore, any reference to ‘international students’ in the findings should be read as ‘students from the Indian subcontinent.’

### Academic and professional staff-initiated actions to alleviate cross-cultural challenges

#### (A) clear communication of academic and policy expectations

One of the most important issues identified was subcontinent students’ lack of understanding of the university’s expectations. The participants especially highlighted expectation gaps in the context of course-related activities like study commitment, self-direction, critical analysis and academic conventions and skills. There were several comments from faculty participants indicating that outlining goals and expectations at the beginning of a course or prior to assessment due dates could alleviate this problem:

(…) it is necessary to indicate in Week 1 lectures about how the material is delivered each week, where resources are provided and the assessment tasks and expectations from students (FG2_AS_P7).

To explain expectations in terms of academic conventions, academic staff suggested practical demonstrations of key academic skills, including referencing, paraphrasing and academic writing.

Sometimes I will go into the library and simply give them a research hour. Say, “Here is how we find articles. This is what you need to do.” Sometimes I will show previous assignments. Pop them up and say, “Look. This is how it is structured. This is how the referencing works” (FG1_AS_P2).

This suggestion appears to work well with subcontinent students’ prior educational experience in their home country, where they often received all the information “on a platter,” as opposed to being told what to do and then independently completing the said task (Manikutty et al., 2007, p. 84).

The focus group participants also acknowledged the importance of ongoing expectation management, particularly the need to continually emphasise assessment requirements. Participants suggested that repeating assessment requirements is important for subcontinent students because they generally grew up in a different education system where these types of reminders were commonplace:

At least 2–3 weeks prior to an assessment task’s due date, an explanation about the assessment is provided. This explanation includes how these tasks are relevant to the content covered so far. As these students have little or no exposure to completing assessment tasks in previous units of study in their home country, a discussion on assessment tasks will help them to understand the expectations for each assessment task (FG2_AS_P7).

In the context of different educational cultures, focus group participants supported Marambe et al., (2012) and Newton et al.’s (2021) findings that subcontinent students are also often not familiar or comfortable with student-centred, independent, and the deep learning practices that is expected of students in Australia’s higher education system. Baeten et al., (2010) argue that this transition requires teaching approaches that assist students to adapt to this new approach. Focus group participants recommended that international students receive clear explanations of what student-centred learning entails, coupled with some live demonstrations of key learning practices.

A further academic area in which expectation management for subcontinent students is crucial refers to the issue of academic integrity. Subcontinent students are often not overly familiar with the concept of academic integrity as practised in Australia. As such, academic staff should ensure that expectations around academic integrity are clearly explained, and opportunities to cheat are reduced (FG2_AS_P5; FG3_AS_P1).

Participants stressed the importance of engagement with subcontinent students immediately after or even before they arrived in Australia, as illustrated by the following quote:

Probably, more engagement. You know, that is the first day a student comes to university. What are our expectations, and how are we meeting their expectations? (FG6_PS_P5).

To facilitate pre-arrival expectations, professional staff suggested substituting written materials with videos as ‘video presentations and things that are a little more engaging and accessible rather than a handbook and things like that’ (FG5_PS_P1). However, it emerged that live, synchronous web interaction would be even better than recorded videos:

We want to put on webinars, so we can actually engage with students before they arrive, to help them sort out things like accommodation, help clarify expectations and things like that (FG5_PS_P1).

Given the importance of interpersonal relationships for members of collectivist cultures, it is not surprising that a more personal approach, such as a synchronous webinar with university staff, might be preferable for students from the Indian subcontinent (Shuter et al., 2018). While focus group participants considered the use of cost-effective audio-visual material for engagement to be superior to written content, there is an argument that direct interaction with students instils an even greater sense of importance when communicating expectations. Video material facilitates deep learning provided that the relevance of the material is made clear to learners (Mitra et al., 2010). Therefore, it is unlikely that stand-alone videos will positively impact an international student’s understanding of practical issues such as accommodation unless staff proactively engage with students to emphasise the importance of the information portrayed in the video. Webinars are a more interactive web-based means of visually communicating with students.

#### (B) customising the pedagogic environment

Focus group participants noted that many students from the subcontinent struggled with English and studying the course material in an Australian context. These experiences highlight the broader literature on international students, which point out problems associated with language barriers (Costa, 2019; Carroll & Ryan, 2007; Newton et al., 2021) and challenges relating to the use of local examples (Bird, 2017). To alleviate these challenges, faculty members provided a range of actions that could be introduced to improve the learning environment for subcontinent students. These included contextualising teaching materials to the student’s cultural or country background, creating subtitled videos to supplement course notes, redesigning classrooms and offering language support courses.

a) *Contextualise course delivery to students’ background*: The academic focus groups discussed the value of providing subcontinent students with examples and case studies from their home country. The below quotes exemplify this suggestion by presenting the experiences of academic participants.

A strategy that I have adopted and find works very well with students from the subcontinent when teaching marketing is using examples relevant to the country they come from (FG1_AS_P7).

And I find that my students typically when I am talking to them about innovation and management, the examples drawn from the automotive industry, cars. They are interested in cars. Really interested in cars. They are very interested in phones. Very interested in technology. These things resonate with them (FG1_AS_P2).

*b) Supplementing teaching materials with (captioned) videos*: Several faculty members mentioned that video materials effectively supplement written materials (FG1_AS_P3; FG2_AS_P1). In addition, the literature suggests that video material facilitates deep learning provided that the relevance of the materials (Mitra et al., 2010) and value of the academic offer is made clear to learners (Dicker et al., 2019).

As part of the development of video resources, some focus group participants recommended captioning (initially intended for hearing-impaired students) as a useful source of vocabulary to support subcontinent students’ learning:

I have got all of the videos of the slides with me narrating them. They have been captioned now for hearing. Look at the slides with the captions, because they think, “well, I do not quite understand him, but I have got captions down there” (FG2_AS_P1).

*c) Redesigning classrooms and learning spaces*: The focus groups regularly referred to classroom design that could support subcontinent students’ learning as well as their adjustment to the Australian requirement for academic integrity:

I would like to see ‘pod’ classrooms where we assess collaborative exercises. My experiences of individual class exercises in the assessment are that it is too difficult to prevent the spread of answers around the room given the lab setup (FG4_AS_P5).

In a study of the impact of different computer lab seating arrangements on learning outcomes, Callahan (2004a) refers to a ‘pod’ as a desk with a cluster of four computers facing each other. Callahan concluded that the pod arrangement enhanced student-teacher interactions and facilitated more active engagement in the learning process. One focus group member stressed the importance of seating in open areas to promote out-of-class interactions among students and between students and staff (FG4_AS_P5). Given the importance of interpersonal relationships in collectivist cultures like those in Indian subcontinent countries (Shuter et al., 2018), it would be no surprise if subcontinent students would benefit from such a setup.

*d) English language and bridging courses*: Professional staff expressed strong views on providing enhanced English language and bridging programs for subcontinent students before enrolling in a specific course to address deficiencies in English language skills as identified in Newton et al., (2021). While the provision of such courses is the responsibility of university administrators, the discussion is included here because of its relevance to the pedagogic environment, as such bridging programs would allow students to adjust to the Australian context before starting their actual course:

Those who go through an English program before, they are very well prepared. They are already “kind of introduced” to how the university works as well as the terminology and the expectations (FG8_PS_P1).

There is evidence that pre-program intensive bridging courses enhance academic outcomes and learner satisfaction (Schmid et al., 2012; Teo & Chang, 2018).

### Institutional actions to alleviate cross-cultural challenges

#### (A) peer-to-peer mentoring

Several professional staff commented that the engagement of good students as teaching assistants or peer-to-peer mentors could improve subcontinent students’ learning experience, helping them integrate into the university community. The following quote from an academic staff member exemplifies this notion:

(…) providing a program that is like peer-to-peer mentoring. International students rely a lot on their friends, on their mates, for information, for guidance; it might be useful to have senior students paired up with a junior student to provide guidance and help (FG1_AS_P8).

The literature confirms that mentoring programs provide many benefits to the mentee, mentor, and host institution (e.g., Peregrina-Kretz et al., 2018; Ring, 2015). Peer mentoring and access to information-related social support are highly relevant in the higher education sector and positively impact higher education outcomes (Mishra, 2020).

#### (B) resourcing of skill and mental health support services for subcontinent students

Academic and professional staff focus group participants regularly referred to the benefits derived from academic skills support services provided through a Centre for Academic Learning. Such a centre could take on the task of a live demonstration of key academic skills so that educators can focus their time more on the curriculum. However, given that the offerings of this centre may be extracurricular, subcontinent students may not consider the facilitators as ‘important’ as the teachers/gurus teaching their course (Manikutty et al., 2007; Marambe et al., 2012; Newton et al., 2021) and therefore it is questionable whether this particular cohort of students would make extensive use of such services (Newton et al., 2021).

To a lesser extent, some of the focus group participants also recommended that institutions ensure the appropriate availability of counselling resources for international students as the various challenges they face may result in mental health problems that can be alleviated through counselling support (FG8_PS_P2). Counselling services are needed in the higher education sector to improve students’ relations with their teaching staff, mentors and peers (Mishra, 2020). The literature suggests that many international students face mental health problems due to challenges associated with living in a foreign country, being homesick, and acculturation challenges (Chirkov et al., 2008; Mishra, 2020; Newton et al., 2021).

#### (C) implementing cross-cultural awareness training:

The literature suggests that cross-cultural awareness training performs an important role in assisting academic and professional staff to support international students (e.g., Jacob et al., 2015; Vinther & Slethaug, 2015; Zhang & Dinh, 2017). However, focus group members expressed divergent views on this issue:

Several professional staff members supported cultural awareness training, suggesting that such training would enhance student satisfaction (FG5_PS_P3; FG5_PS_P2). However, it needs to be a ‘continuous process rather than a one-off training’ (FG2_AS_P6). In addition, it should be based on identified issues relevant to the staff member’s work and contain an overview of subcontinent students’ cultural traits:

I definitely think there is room for more cultural awareness training across the board, but also depending on which business unit and what their role is, we need to define what that is (FG5_PS_P1).

However, there were some negative opinions among focus group participants regarding the implementation of cross-cultural training. This negativity did not reflect an unwillingness on the part of those participants to better play their role in facilitating student transition (Hachfeld et al., 2015). Instead, it referred to their assessment of their own extensive experience of working with subcontinent students, as well as pragmatic workload considerations:

I think, there is always room for more, definitely, as far as our role to support students is concerned. I will probably speak about just the student experience staff, we have got to define exactly what it is [cultural awareness] so we [better understand what we] were meant to be doing at the moment. We are administrators, so if that is all we are meant to be doing, we have probably got enough training (FG5_PS_P1).

The variety of training requirements presented by participants in the focus groups, alongside the literature’s overly generic use of ‘cross-cultural training,’ without detailing what this entails, suggests that offering a one-size-fits-all training program may not work. Moreover, the negative voices indicated that the provision of cross-cultural training to university staff requires careful assessment of the cross-cultural expertise of each staff member and the specific roles they fill. Since university staff in Australia are culturally diverse (Welch, 2020), a blanket approach to cross-cultural training may serve to alienate and offend participants if training pre-assessment is not conducted.

#### (D) reform student recruitment practices

Both academic and professional staff expressed concerns that many subcontinent students’ primary focus was to achieve an immigration outcome (i.e., permanent residency) rather than an academic outcome. (FG5_PS_P1). There were also comments relating to the difficulty of getting subcontinent students to understand the conditions of their student visas, especially when it came to minimum class attendance requirements. For example:

You are on a student visa. You have to study five days a week. They [students] have to be enrolled full-time (FG6_PS_P2).

Some participants attributed the blame for ‘selling’ Australian education as a ticket to a permanent residency status to overseas agents:

We need to overcome the visa mentality, which I know a lot of our students have, they are here seeking a visa, and that is really a lot of the students. We need to improve our recruitment techniques move away from the selling of a visa, which is, unfortunately, [what] a lot of our agent networks [do]. That is what they do (FG3_AS_P2).

Recruiting subcontinent students seeking immigration opportunities via university enrolment was a significant finding of the focus groups. It would also seem that subcontinent students tend to pursue other classes of visas after arriving in Australia, as confirmed in Birrell’s (2019 p. 10) study of international students and their impact on net migration. Focus group participants suggested that a greater effort needs to be made by universities to ensure overseas agents and recruiters are providing the right information to potential students, including the temporary nature of their legal status in Australia and the requirement to observe the conditions of their student visas.

## Conclusions

This paper has focused on the perceptions and experiences of academic and professional staff in the context of alleviating cross-cultural challenges faced by students from the Indian subcontinent. The study addresses two related gaps in the current literature, which ignores the cross-cultural challenges faced by students from the Indian subcontinent and neglects the staff perspective on how they can alleviate such challenges. Given the large and growing numbers of subcontinent students enrolled in the Australian higher education system, this study is timely and important.

To address its purpose, this study sought staff perceptions to the research question: - *Based on the experience of staff working with subcontinent students, what initiatives can be implemented in universities to help alleviate the cross-cultural challenges faced by Indian subcontinent students?* Emerging suggestions can be classified into initiatives that academic staff and university administrators could introduce. One of the most important initiatives for academic staff, professional staff, and university administrators is to focus extensively on managing subcontinent students’ expectations pre and post-arrival, in the context of academic conventions, the Australian education system, support services (e.g., accommodation) and visa requirements. The latter would require universities to ensure overseas agents provide correct information to prospective students. Another helpful initiative would be to customise the pedagogic environment within which subcontinent students learn. This may include contextualising course delivery through real-life examples of relevance to the students’ backgrounds, supplementing teaching materials with captioned videos, and facilitating the development of interpersonal relationships through classroom layout.

In terms of institutional initiatives, staff recommended that peer-to-peer mentoring programs and programs to help improve students’ academic and language skills could be of particular value to students from the collectivist Indian subcontinent. The provision of cross-cultural awareness training for academic and professional staff was met with mixed enthusiasm.

This study has practical and policy implications for Australian universities (and others globally) in providing cross-cultural training to academic and professional staff to better understand and address the cross-cultural concerns faced by students from the Indian subcontinent. Theoretically, this paper highlights the importance of cross-cultural issues in the higher education context as perceived by professional and academic staff at the forefront of the interactions with students from diverse cultural backgrounds. In line with existing research (Newton et al., 2021; Ross, 2020), addressing the identified cross-cultural issues would assist Australian universities in attracting students from subcontinent backgrounds back to Australia post-COVID 19. In addition, future research can explore how the challenges identified in this study are relevant to help improve education service delivery to international students from other countries such as China, Nepal, Vietnam, Malaysia, and Indonesia, from which Australia has a high student inflow (Department of Education, Skills and Employment, Australian Government 2020; Ross 2020; Welch, 2020).

As with any study, this one is not without limitations. First, the findings from this study should not be generalised to the entire international student body, as the focus was on students from the Indian subcontinent. Similar studies could be conducted to understand the actions taken by academic and professional staff to alleviate the challenges faced by students from other regions, such as Latin America, Europe and China. Second, this paper follows Atri and Sharma’s (2006) approach of treating students from the Indian subcontinent as a homogeneous group because they share a very similar culture. Still, considering the size and diversity of the Indian subcontinent, such an approach may reflect an oversimplified research design. Third, although a sufficiently large number of staff participated in the focus groups, the study was conducted at one Australian university, and therefore the findings should be treated accordingly. Finally, since only staff views were explored, future studies could consider exploring the perspectives of students from the subcontinent.

## Data Availability

Owned by the researchers.
